# Risk factors for antimicrobial resistance in patients with *Escherichia coli* bacteraemia related to urinary tract infection

**DOI:** 10.1016/j.infpip.2022.100248

**Published:** 2022-09-03

**Authors:** James Balfour, Mabel Barclay, Janathan Danial, Carol Philip, Meghan Perry, Michelle Etherson, Naomi Henderson

**Affiliations:** Clinical Infection Research Group, NHS Lothian Infection Service, Western General Hospital, United Kingdom

## Abstract

**Introduction:**

NHS Lothian policy has recently changed to avoid first-line use of trimethoprim for uncomplicated urinary tract infections (UTI) in patients with risk factors for trimethoprim resistance, in line with national guidance. This study aimed to identify risk factors for antimicrobial resistance in *Escherichia coli* bacteraemia related to UTI.

**Methods:**

A retrospective cohort study of 687 patients with *E. coli* bacteraemia related to UTI in NHS Lothian from 01/02/18 to 29/02/20 was undertaken. Demographics and comorbidities were collected from electronic patient records. Community prescribing and microbiology data were collected from the prescribing information system and Apex. Univariate and multivariate analysis was undertaken using RStudio to analyse trimethoprim, gentamicin and multi-drug resistance (MDR).

**Results:**

Trimethoprim resistance was present in 282/687(41%) of blood culture isolates. MDR was present in 278/687(40.5%) isolates. Previous urinary trimethoprim resistant *E. coli* was a significant risk factor for both trimethoprim resistance (OR 9.44, 95%CI 5.83–15.9) and MDR (OR 4.81, 95%CI 3.17–7.43) on multivariate modelling. Trimethoprim prescription (OR 2.10, 95% CI 1.33–3.34) and the number of community antibiotic courses (OR 1.19, 95%CI 1.06–1.35) were additional risk factors for trimethoprim resistance. Multiple independent risk factors were also identified for trimethoprim resistance, MDR and gentamicin resistance.

**Discussion:**

This study showed a high prevalence of trimethoprim resistance and MDR in patients with *E. coli* bacteraemia related to UTI. This supports the withdrawal of trimethoprim from first-line treatment of UTIs in patients with risk factors for trimethoprim resistance. It has also identified risk factors for MDR in *E. coli* bacteraemia.

## Introduction

Antimicrobial resistance (AMR) is a significant and increasing problem worldwide. Urinary tract infections (UTI)s are a common cause of morbidity and mortality with severe complications including bacteraemia and sepsis. *Escherichia col*i is the most common organism isolated from urinary samples in Scotland, England and Europe [[1], [2], [3]]. *E. coli* bacteraemia is strongly associated with UTI and accounts for the majority of Gram-negative bacteraemias in Scotland [4]. AMR is highly prevalent in urinary *E. coli* and *E. coli* bacteraemia in Scotland [4,5]. *E. coli* bacteraemia is associated with 30-day all-cause mortality from 8-18.2% in hospital inpatients [[6], [7], [8]] and associated AMR leads to less effective management [7,[9], [10], [11]].

*E. coli* was isolated from 115,844 urine samples and 4,206 blood cultures across Scotland in 2020 [1]. The incidence of *E. coli* bacteraemia was 77 per 100,000 population [1]. This has been broadly stable over the last five years [1,4]. The high incidence of trimethoprim resistance in urinary *E. coli* led to NICE recommending avoidance in patients at risk of resistance. Risk factors identified included hospital inpatients, nursing or care home residents, over 65-year-olds, trimethoprim prescription in the previous three months, or trimethoprim resistant organisms in the previous three urine cultures, over the previous 12 months [12]. NHS Lothian guidelines for community antibiotic prescribing changed to reflect this after the data for this study was collected. In 2019, the (UK) Government set the target of a 50% reduction in Gram-negative bacteraemias by 2023/2024. [4] This study aimed to examine the risk factors for AMR and MDR in *E. coli* bacteraemia related to UTI to allow the review and optimisation of local empirical antimicrobial prescribing guidelines and subsequently reduce the number of *E. coli* bacteraemias related to UTI.

## Methods

This was a retrospective cohort study of all patients with *E. coli* bacteraemia associated with UTI in blood cultures across NHS Lothian from 01/02/2018 to 29/02/2020. In total, 710 *E. coli* bacteraemia isolates related to UTI were identified. Repeat isolates and paediatric cases were removed leaving 687 cases. Clinical data, demographics and comorbidities were drawn from Trak (online patient records) and microbiology data from Apex (laboratory systems). Demographic data included age, gender and nursing home residence. Comorbidities included diabetes mellitus, previous urological surgery and long-term catheterisation. Urinary *E. coli* isolates in the previous 12 months were analysed. Community prescribing data was collected over 6 months before the bacteraemia from the prescribing information system (PIS).

Data manipulation, univariate and multivariate analyses were carried out in RStudio version 4.1.0. Multivariate models were evaluated using the Akaike information criterion (AIC) with a backwards stepwise method using the ‘MASS’ package [13]. Collinearity between risk factors was allowed for.

All isolates received standard sensitivity testing using VITEK® 2 automated broth microdilution cards N381 (blood cultures) and N382 (urines) and categorised using EUCAST breakpoints v8.0, v8.1 and v9.0 [14]. Whilst there is no standard bacteraemia breakpoint for trimethoprim resistance, this was extrapolated from urinary breakpoints as a minimum inhibitory concentration of >4mg/L. Gentamicin is the first-line treatment of upper UTI and sepsis secondary to urinary source within NHS Lothian guidelines, so was also analysed [15]. There is no globally accepted definition for MDR and extensively drug resistant (XDR) Enterobacteriaceae. The ECDC definition [16] is not suited for routine clinical use as it utilises 17 different antimicrobial categories and contains agents, including entire classes, which are not routinely tested for in Scotland. For this reason, the Canadian system for describing MDR and XDR Enterobacteriaceae, described by German *et al.* (2018), currently under proposal for use in NHS Lothian, was used to categorize MDR and XDR isolates [17]. Isolates were described as MDR if they were resistant to three or four of the six groups of antimicrobials listed below. XDR was defined as greater than four of the six groups. Antimicrobial sensitivities were divided into the following groups: tobramycin/gentamicin, piperacillin-tazobactam, imipenem/meropenem, cefotaxime/ceftriaxone/ceftazidime, ciprofloxacin and trimethoprim-sulfamethoxazole [17]. In our analysis trimethoprim was used to indicate trimethoprim-sulfamethoxazole susceptibility. Intermediate resistance, as per EUCAST breakpoints v12.0, was included with resistant isolates as these antibiotics would be avoided in practice [14].

## Results

### Demographics

**Figure 1:**
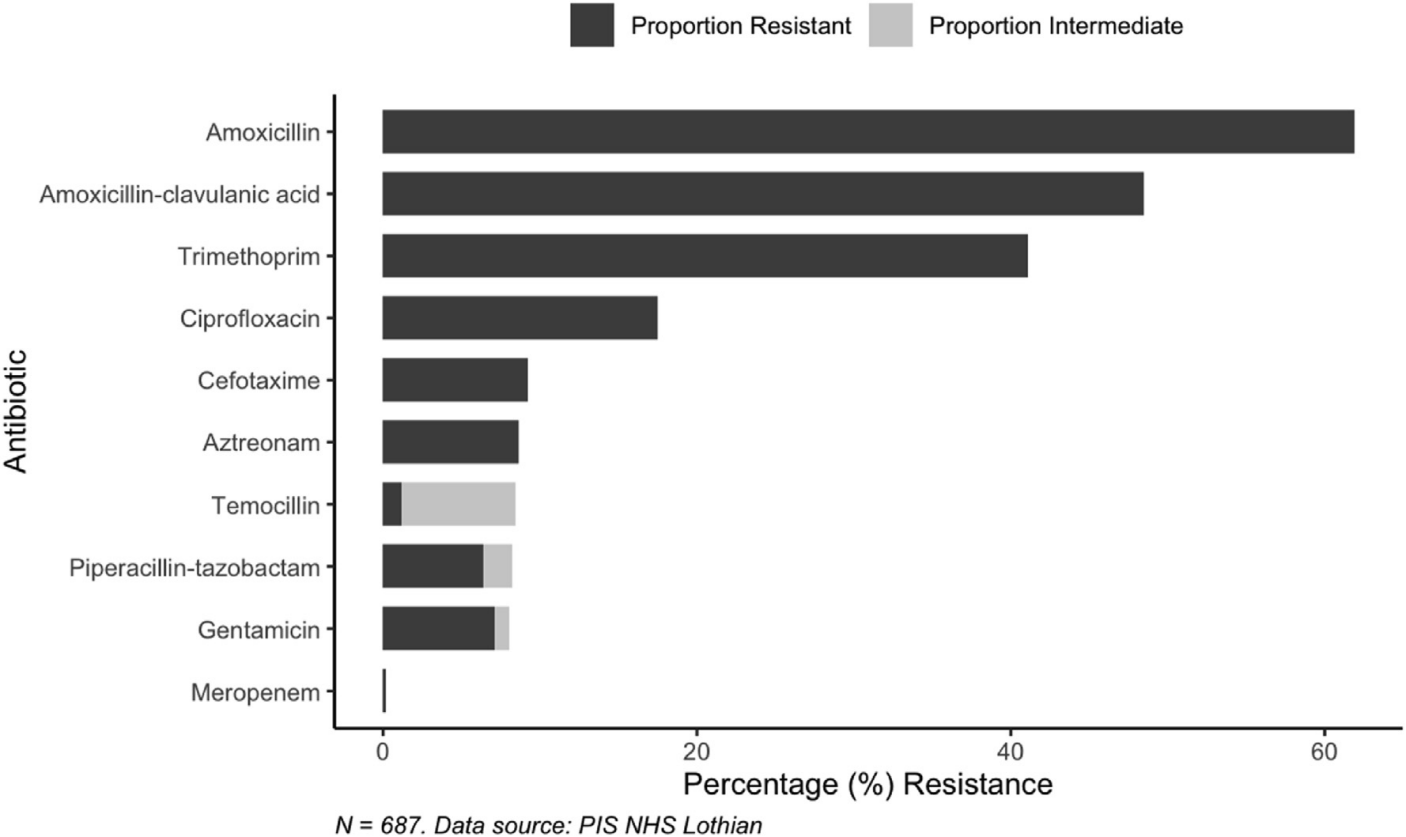
The percentage of *E. coli* bacteraemia isolates demonstrating resistance to antimicrobials in 687 isolates related to UTI in NHS Lothian.

687 patients with UTI related *E. coli* bacteraemia were examined with a mean age of 71.3 years. There were 407 (59.2%) female patients. Nursing home residents made up 47/687 (6.84%). Insulin-dependent diabetes was present in 50/687 (7.28%) patients. Long term catheters were present in 162/687 (23.58%) patients. Previous urological surgery had occurred in 94/687 (13.68%) patients. Prophylactic antibiotic prescription in the community occurred in 33/687 (4.80%) patients. The proportion of isolates with AMR to commonly used antibiotics was measured across the cohort (Figure 1).

### Trimethoprim resistance

Trimethoprim resistance was present in 282/687 (41.05%) *E. coli* bacteraemia isolates (Figure 1). Univariate analysis revealed significant risk factors to be community trimethoprim prescription (OR 2.71, 95%CI 1.87–3.96), the number of trimethoprim courses (OR 1.66, 95%CI 1.33–2.12), community antibiotic prescription (OR 2.05, 95%CI 1.51–2.81), the number of antibiotic courses (OR 1.24, 95%CI 1.13–1.37), previous urinary trimethoprim resistant *E. coli* (OR of 8.56, 95%CI 5.49–13.80), and community prophylactic antibiotic prescription (OR of 3.03, 95%CI 1.48–6.59) (Table I).

**Table I:** Univariate and multivariate analysis of risk factors for trimethoprim resistance in *E. coli* bacteraemia related to UTI in 687 patients in NHS Lothian

Risk factors for trimethoprim resistance								
Characteristic	N	n	Univariate analysis			Multivariate analysis		
			OR^1^	95% CI^2^	*P*-value	OR^1^	95% CI^2^	*P*-value
Community trimethoprim prescription^a^	687	146	2.71	1.87, 3.96	<0.001	2.10	1.33, 3.34	0.002
Community antibiotic prescription^a^	687	357	2.05	1.51, 2.81	<0.001			
Age	687		1.01	1.00, 1.02	0.20			
Gender	687	280^3^	1.19	0.88, 1.62	0.30			
Nursing home resident	687	47	1.41	0.78, 2.56	0.30			
Insulin dependent diabetes mellitus	687	50	1.36	0.76, 2.42	0.30			
Upper UTI/Pyelonephritis	687	173	0.80	0.56, 1.13	0.20			
Long term catheter	687	162	1.32	0.93, 1.89	0.12			
Previous urological surgery	687	94	1.13	0.73, 1.75	0.60			
Previous trimethoprim resistant urinary *E. coli*	687	134	8.56	5.49, 13.8	<0.001	9.44	5.83, 15.9	<0.001
Community nitrofurantoin prescription^a^	687	73	1.37	0.84, 2.23	0.20	0.28	0.14, 0.56	<0.001
Community prophylactic antibiotic prescription^a^	687	33	3.03	1.48, 6.59	0.003			
Community antibiotic prescription except trimethoprim^a^	687	211	1.04	0.75, 1.44	0.80			
Number of trimethoprim courses^a^	687		1.66	1.33, 2.12	<0.001			
Number of antibiotic courses^a^	687		1.24	1.13, 1.37	<0.001	1.19	1.06, 1.35	0.004

^1^OR = Odds ratio, ^2^Ci= Confidence interval, ^3^Gender n= male.

a Prescribing data from 6 months prior to bacteraemia.

**Figure 2:**
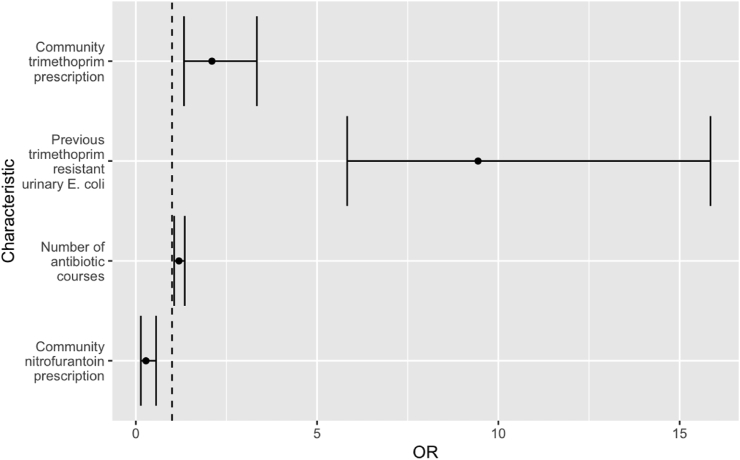
Forest plot demonstrating odds ratio with 95%CI for predictors of trimethoprim resistance in *E. coli* bacteraemia related to UTI following multivariate modelling of 687 isolates from NHS Lothian. (OR = Odds ratio).

Multivariate analysis showed that previous trimethoprim resistant urinary *E. coli* (OR 9.44, 95%CI 5.83–15.9), community trimethoprim prescription (OR 2.10, 95% CI 1.33–3.34) and the number of antibiotic courses (OR 1.19, 95%CI 1.06–1.35) were significant risk factors for trimethoprim resistance in E. coli bacteraemia. Nitrofurantoin prescription was negatively associated with trimethoprim resistance (OR 0.28, 95%CI 0.14–0.56) (Table I, Figure 2).

### MDR

MDR was present in 278/687 (40.4%) isolates. Trimethoprim resistance was present in 226/278 (81.3%) of MDR isolates. Univariate analysis revealed significant risk factors for MDR were community trimethoprim prescription (OR 1.96, 95%CI 1.35–2.83), the number of trimethoprim courses (OR 1.50, 95% CI 1.22–1.88), community antibiotic prescription (OR 1.87, 95%CI 1.37–2.55), the number of antibiotic courses (OR 1.26, 95%CI 1.15–1.39), previous trimethoprim resistant urinary *E. coli*, (OR 5.39, CI 3.58–8.27), community nitrofurantoin prescription (OR 2.17, 95%CI 1.33–3.57) and community prophylactic antibiotic therapy (OR 3.60, 95%CI 1.73–8.03) (Table II).

**Table II:** Univariate and multivariate analysis of risk factors for MDR in *E. coli* bacteraemia related to UTI in 687 patients in NHS Lothian

Risk factors for MDR								
Characteristic	N	n	Univariate analysis			Multivariate analysis		
			OR^1^	95% CI^2^	*P*-value	OR^1^	95% CI^2^	*P*-value
Community trimethoprim prescription^a^	687	146	1.96	1.35, 2.83	<0.001			
Community antibiotic prescription^a^	687	357	1.87	1.37, 2.55	<0.001			
Age	687		1.01	1.00, 1.02	0.050			
Gender	687	280^3^	1.12	0.82, 1.53	0.5			
Nursing home resident	687	47	1.59	0.87, 2.88	0.13			
Insulin dependent diabetes mellitus	687	50	1.07	0.59, 1.91	0.8			
Upper UTI/Pyelonephritis	687	173	0.77	0.54, 1.10	0.2			
Long term catheter	687	162	1.28	0.90, 1.83	0.2			
Previous urological surgery	687	94	1.49	0.96, 2.31	0.073			
Previous trimethoprim resistant urinary *E. coli*	687	134	5.39	3.58, 8.27	<0.001	4.81	3.17, 7.43	<0.001
Community nitrofurantoin prescription^a^	687	73	2.17	1.33, 3.57	0.002			
Community prophylactic antibiotic prescription^a^	687	33	3.60	1.73, 8.03	<0.001			
Community antibiotic prescription except trimethoprim^a^	687	211	1.21	0.87, 1.67	0.3			
Number of trimethoprim courses^a^	687		1.50	1.22, 1.88	<0.001			
Number of antibiotic courses^a^	687		1.26	1.15, 1.39	<0.001	1.19	1.09, 1.31	<0.001

^1^OR = Odds ratio, ^2^Ci= Confidence interval, ^3^n= male.

a Prescribing data from 6 months prior to bacteraemia.

Multivariate analysis showed that previous trimethoprim resistant urinary E. coli (OR 4.81, 95%CI 3.17–7.43) and the number of community antibiotic courses were significant risk factors for MDR (Table II).

Only 8/687 cultures were extremely drug-resistant (XDR) therefore no additional analyses were undertaken.

### Gentamicin resistance

Gentamicin resistance was present in 55/687 (8.0%) isolates (Figure 1). Univariate analysis revealed risk factors for gentamicin resistance to include community antibiotic prescription (OR 1.83, 95%CI 1.04–3.32), age (OR 1.02, 95%CI 1.00–1.05), long term catheterisation (OR 1.97, 95%CI 1.08–3.48) and previous urinary trimethoprim resistant *E. coli* (OR 7.22, 95%CI 4.08–13.00) (Table III). No multivariate model demonstrated a good fit for the gentamicin data (Table IV).

**Table III:** Univariate analysis of risk factors for gentamicin resistance in *E. coli* bacteraemia related to UTI in 687 patients in NHS Lothian

Risk factors for gentamicin resistance					
Characteristic	N	n	Univariate analysis		
			OR^1^	95% CI^2^	*P*-value
Community trimethoprim prescription^a^	687	146	1.29	0.66, 2.39	0.4
Community antibiotic prescription^a^	687	357	1.84	1.04, 3.33	0.039
Age	687		1.02	1.00, 1.05	0.024
Gender	687	280^3^	1.23	0.70, 2.14	0.5
Nursing home resident	687	47	0.49	0.08, 1.66	0.3
Insulin dependent diabetes mellitus	687	50	1.64	0.60, 3.77	0.3
Upper UTI/Pyelonephritis	687	173	0.56	0.25, 1.11	0.12
Long term catheter	687	162	1.97	1.09, 3.49	0.022
Previous urological surgery	687	94	1.87	0.91, 3.59	0.071
Previous trimethoprim resistant urinary *E. coli*	687	134	7.23	4.08, 13.0	<0.001
Community nitrofurantoin prescription^a^	687	73	1.74	0.77, 3.56	0.2
Community prophylactic antibiotic prescription^a^	687	33	0.35	0.02, 1.66	0.3
Community antibiotic prescription except trimethoprim^a^	687	211	1.56	0.88, 2.74	0.12
Number of trimethoprim courses^a^	687		0.91	0.58, 1.27	0.6
Number of antibiotic courses^a^	687		1.06	0.93, 1.19	0.3

^1^OR = Odds ratio, ^2^Ci= Confidence interval, ^3^n= male.

a Prescribing data from 6 months prior to bacteraemia.

**Table IV:** Summary of risk factors for antibiotic resistance in E. coli bacteraemia related to UTI

Summary table	
**Risk factors for trimethoprim resistance identified through multivariate analysis**	***OR***^***1***^
Previous trimethoprim resistance in urinary E. coli isolate within 12 months	9.44
Community trimethoprim prescription	2.10
Cumulative community antibiotic prescribing	1.19
**Risk factors for MDR identified through multivariate analysis**	***OR***^***1***^
Previous trimethoprim resistance in urinary E. coli isolate within 12 months	4.81
Cumulative community antibiotic prescribing	1.19

OR^1^= Odds ratio.

## Discussion

The demographics and resistance profile of the cohort were concordant with Scottish, English and European surveillance data [[1], [2], [3]]. This study showed that previous urinary trimethoprim resistant *E. coli* was the most significant risk factor for trimethoprim resistance and MDR in *E. coli* bacteraemia. Trimethoprim resistance was also associated with trimethoprim prescription and the number of antibiotic courses prescribed. MDR was associated with the number of community antibiotic courses prescribed. This agrees with the multiple studies demonstrating community antibiotic prescribing is linked to AMR in *E. coli* urinary and blood isolates [5,18,19]. This study mainly concurs with previous research into risk factors for resistance in urinary *E. coli* isolates completed by Malcolm *et al.* (2017) which identified risk factors for AMR and MDR in urinary *E. coli* across 40,984 isolates in Scotland [5]*.* Malcolm *et al.* demonstrated trimethoprim, nitrofurantoin and cumulative antibiotic use to be risk factors for AMR and MDR [5]. However, the present study showed that nitrofurantoin prescription was negatively associated with trimethoprim resistance. This relationship requires further exploration, particularly as nitrofurantoin use may increase with the change in local guidelines.

In the present study, clinical factors including age, diabetes mellitus, long-term catheterisation, and previous urological surgery were assessed. Age and long-term catheterisation were independent risk factors for gentamicin resistance. Prophylactic antibiotic therapy in the community was independently linked to both trimethoprim resistance and MDR. In a recent study, Aliabadi *et al.* (2021) analysed risk factors for AMR in *E. coli* bacteraemia in 175,147 patients from English national surveillance data. Increasing age and regional deprivation were associated with AMR in community-acquired *E. coli* bacteraemia [20].

In the present study, univariate analysis revealed a correlation between trimethoprim resistance in urinary *E. coli* and gentamicin resistance in *E. coli bacteraemia*. This effect, alongside the failure of multivariate modelling for gentamicin resistance, may be due to the small sample size. However, this link should be explored further to allow the optimisation of community prescribing.

The strengths of this study include the analysis of blood culture isolates, guaranteeing the significance of the infection. Some urinary isolates may reflect asymptomatic bacteriuria. However, it has been suggested that AMR bacteria may be more likely to progress to bacteraemia [10]. This study analysed resistance to trimethoprim, gentamicin and MDR, rather than a single antimicrobial.

The limitations of this study include a lack of inpatient prescribing data. Patients may have received antibiotics as an inpatient prior to the development of the bacteraemia. Temporal data from the community prescribing was not collected therefore the length of time from prescription to bacteraemia could not be analysed.

## Conclusion

Trimethoprim resistance and MDR are prevalent in *E. coli* bacteraemia associated with UTI. The most significant risk factor for trimethoprim resistance and MDR was previous urinary trimethoprim resistant *E. coli*. Community trimethoprim prescription and cumulative community antibiotic courses were also associated with trimethoprim resistance. Many factors, particularly community antibiotic prescribing, were independently linked to AMR and MDR. This study supports the move away from first-line trimethoprim use for UTIs in patients with risk factors for resistance. Since the data collection period, NHS Lothian has moved away from first-line trimethoprim use in patients at risk of resistance. This data should be considered to further optimise empirical prescribing guidelines in the hope it may lead to a reduction in *E. coli* bacteraemias.

## Conflict of interest statement

No conflict of interest was identified.

## Funding statement

This research did not receive any specific grant from funding agencies in the public, commercial, or not-for-profit sectors

## Credit author statement

**James Balfour:** Conceptualisation, methodology, investigation, writing-original draft, visualisation, project administration.

**Mabel Barclay:** Software, formal analysis, data curation.

**Janathan Danial:** Data curation.

**Carol Philip:** Data curation.

**Meghan Perry:** Conceptualisation, Writing-review and editing, supervision.

**Michelle Etherson:** Conceptualisation, writing-review and editing, supervision.

**Naomi Henderson:** Conceptualisation, writing-review and editing, supervision.
